# Pneumomediastinum and Pneumopericardium as Uncommon Complications of COVID-19 Infection: A Review Article

**DOI:** 10.7759/cureus.30244

**Published:** 2022-10-13

**Authors:** Amjad S Alsulaihebi, Murouj Almaghrabi, Muath M Alqarni, Amal Al-Doboke, Abdulmonim Alqasim

**Affiliations:** 1 Department of Medicine and Surgery, College of Medicine, Umm Al-Qura University, Makkah, SAU; 2 Department of Physiology, College of Medicine, Umm Al-Qura University, Makkah, SAU

**Keywords:** coronavirus-2, severe acute respiratory syndrome, coronavirus disease 2019, uncommon complication, air leak syndrome, barotrauma

## Abstract

There is an emerging body of literature describing an increasing incidence of pneumomediastinum and, to a lesser extent, pneumopericardium as a complication of COVID-19. However, the literature lacks information regarding patients’ characteristics and a general view of this unusual condition. The purpose of this paper is to summarize the current literature on this phenomenon. In this study, we summarize the risk factors/etiology, imaging modalities, management, and prognosis of known cases in the literature. In total, 48 articles were included in the study, ranging from case reports to case series. Most patients were male (83.3%). The overall mortality rate was 27.1% and the recovery rate was 62.5%.

## Introduction and background

Late in 2019, a new respiratory infectious disease emerged throughout the world, which was then called coronavirus disease 2019 (COVID-19). COVID-19 is caused by severe acute respiratory syndrome coronavirus 2 (SARS-CoV-2) [1]. Human-to-human transmission can occur from aerosols or during medical procedures [2]. Symptoms may occur between two days to two weeks after being exposed to the virus. The most common symptoms are fever, cough, shortness of breath, fatigue, headache, loss of taste or smell, sore throat, nasal congestion, runny nose, nausea or vomiting, and diarrhea [3].

The severity varies from asymptomatic to severe and life-threatening [4]. Serious complications may develop such as pneumothorax [5], pneumopericardium, and pneumomediastinum as reported by Hazariwala [6]. Pneumomediastinum refers to the presence of air in the mediastinum. In spontaneous pneumomediastinum (SPM), there is no known cause, while the etiology in secondary pneumomediastinum is identified [7]. Pneumopericardium occurs when air enters the pericardial sac through a defect near the ostium of the pulmonary veins [8]. This review is intended to provide information on the reports of pneumomediastinum and pneumopericardium that developed in patients with COVID-19 infection. This study examined the risk factors, possible etiologies, imaging modalities, management, prognosis, and characteristics of reported cases.

## Review

Methods

An extensive literature review was conducted using PubMed, EMBASE, and Google Scholar databases to find all relevant cases. Articles that were published from inception to 1 December 2021 were included in the review. The obtained search strategy comprises the following keywords and MeSH terms "COVID-19", "COVID-19", "SARS-CoV-2", "SARS-CoV-2", "SARS-CoV", "novel coronavirus", "pneumopericardium", "PP", "pneumomediastinum", "SPM", and "mediastinal emphysema". Animal studies and non-English investigations were excluded from our search.

The analysis of the current study was conducted using IBM SPSS version 23 for Mac (IBM Corp., Armonk, NY), in which patients’ demographical data, past medical history (known comorbidities), intubation requirement, diagnosis, radiological modality, and outcomes were calculated. Descriptive analysis was used for presenting the data; categorical variables were demonstrated as frequencies and percentages, while continuous variables were demonstrated as mean ± standard deviation.

Results

A total of 48 articles have been found reporting cases of pneumomediastinum and pneumopericardium in patients with COVID-19 (Table 1). The majority of cases were reported among male patients. The minimum age reported was 17 years, and the maximum was 82 years (mean 55.3 ± 14.5). Intubation was not required in more than half of the patients. The outcome of the patients varied, in which 31.25% of the cases died, while 64.58% of them achieved recovery. (Mean recovery time was 19.8 ± 13.5 days.)

**Table 1:** Case reports of pneumomediastinum and pneumopericardium developed in patients with COVID-19 infection. CPAP, continuous positive airway pressure; CT, computed tomography; CXR, chest x-ray; DM, diabetes mellitus; ECMO, extracorporeal membrane oxygenation; GERD, gastroesophageal reflux disease; HFNC, high-flow nasal cannula; HRCT, high-resolution chest tomography; HTN, hypertension; IV, intravenous; LMWH: low-molecular-weight heparin; NA, no available data; NIV, non-invasive ventilator; NKC, no known comorbidities; NRM, non-rebreathing mask; PM, pneumomediastinum; PP, pneumopericardium; PTX, pneumothorax; SOB, shortness of breath; SLE, systemic lupus erythematosus.

No	Reference	Age (years)	Sex	Known comorbidities	Clinical symptoms at presentation	Intubation requirement	Diagnosis	Radiological modality	COVID-19 management	PM/PP management	Outcome
1	Singh, et al. [9]	33	M	NKC	13 days of fever, body aches, SOB, cough, headache, and chest pain	No	PM and PP	Chest CT	Oxygen via nasal cannula, oral azithromycin	Conservative	Recovered after 6 days
2	Li, et al. [10]	68	M	Hypertension, GERD, and acute anuric renal failure	2 weeks of fever, chills, cough, and SOB	Yes, before diagnosis	PM and PP	CXR and chest CT	NA	Conservative	Recovered after 14 days
3	Hazariwala, et al. [6]	57	F	Asthma, HTN, and obesity	17 days of severe cough and progressive SOB	Yes, after diagnosis	PM and PP	CXR and chest CT	HFNC, hydroxychloroquine, zinc, azithromycin, methylprednisolone, LMWH	ECMO	Died after ~ 30 days
4	Hazariwala, et al. [6]	55	M	Asthma, DM, HTN, hyperlipidemia, obesity, smoking	Cough, fevers, and progressive dyspnea	Yes, after diagnosis	PM and PP	CXR and chest CT	HFNC, hydroxychloroquine, zinc, azithromycin, methylprednisolone	Conservative	Died after 50 days
5	Ghods, et al. [11]	82	M	NA	Dyspnea, cough, fever, and malaise	No	PM	CXR and chest CT	NA	Echocardiography-guided catheter insertion	Died after 2 days
6	Sahu, et al. [12]	61	M	HTN	7 days of fever and SOB	Yes, after diagnosis	PM and PP	CXR	Tocilizumab	Conservative	Died after ~ 7 days
7	Rashedi, et al. [13]	55	M	Marginal B-cell lymphoma and mildly overweight	15 days of dry cough, myalgia, and SOB	Yes, after diagnosis	PM and PP	CXR and chest CT	NIV, broad-spectrum antibiotics, remdesivir, methylprednisolone	Conservative	Died after 8 days
8	Scacciavillani, et al. [14]	61	M	NKC	Fever and worsening dyspnea	Yes, before diagnosis	PM and PP	CXR and chest CT	Two prone-positioning sessions, and a tracheostomy on day 14	Conservative	Recovered after 44 days
9	Behzadnia, et al. [15]	24	F	NKC	Fever, myalgia, fatigue, body aches, and headache	No	PM and PP	Chest CT	NIV, dexamethasone, melatonin, LMWH, famotidine, vitamin D3, methylprednisolone, CytoSorb sessions, meropenem, Targocid, vitamin C, aspirin	Conservative	Recovered after 14 days
10	Baburao, et al. [16]	62	F	DM	2 days of cough, fever, and SOB	Yes, after diagnosis	PM and PP	CXR and chest CT	IV remdesivir, IV methylprednisolone, IV piperacillin, and tazobactam	Conservative	Died following multi-organ failure
11	Bistre, et al. [17]	70	F	NKC	7 days of SOB, palpitations, and fever	Yes, after diagnosis	PP	CXR	BiPap	Conservative	Died after 21 days
12	Pimenta, et al. [18]	54	M	NA	14 days of SOB, fever, and fatigue	Yes, before diagnosis	PM and PP	CXR and chest CT	Antibiotics (not specified)	Conservative	Recovered after 54 days
13	Polistina, et al. [19]	70	M	HTN, resolved pericarditis, and Parkinson’s disease	7 days of fever, chills, and SOB	No	PM and PP	HRCT chest, chest CT	HFNC, IV corticosteroids, and remdesivir	Conservative	Recovered after ~22 days
14	Elhakim, et al. [20]	63	M	DM and HTN	2 days of SOB, fever, and fatigue	No	PM	CXR and chest CT	Supplemental oxygen, ceftriaxone, azithromycin, methylprednisolone, LMWH, and remdesivir	Conservative	Recovered after ~44 days
15	Khan, et al. [21, 22]	17	F	Drug abuse	3 days of abdominal pain, nausea, vomiting, diarrhea	No	PM and PP	CXR and chest CT	Normal saline IV, ketorolac, famotidine, and ondansetron	Conservative	Recovered after 7 days
16	Kipourou, et al. [23]	62	M	NKC	9 days of progressive SOB	No	PM	CXR and chest CT	HFNC, remdesivir, IL-1 receptor antagonist, and methylprednisolone	Conservative	Recovered after 18 days
17	Kafle, et al. [24]	44	M	NKC	5 days of fever, cough, and 2 days of SOB	Yes, before diagnosis	PM	HRCT chest, CT chest	Antibiotics, remdesivir, dexamethasone, LMWH, antihistamines, and antipyretics	Conservative	Recovered after 28 days
18	Volpi, et al. [25]	52	M	DM and asthma	Dyspnea, fever, and cough	Yes, before diagnosis	PM and PP	CXR and chest CT	CPAP, co-amoxiclav and doxycycline, epoprostenol, and meropenem	Conservative	Recovered
19	Volpi, et al. [25]	68	M	HTN and hypercholesterolemia	Dyspnea, myalgia, and cough	Yes, before diagnosis	PM	CXR and chest CT	CPAP, co-amoxiclav, and doxycycline	Conservative	Recovered
20	Volpi, et al. [25]	66	M	HTN, obesity, and chronic kidney disease	Fever and acute confusion	Yes, before diagnosis	PP	CXR	CPAP and antibiotics (not specified)	Conservative	Recovered
21	Suresh, et al. [26]	66	M	HTN, chronic kidney disease, and migraine	Cough and SOB	Yes, after diagnosis	PM and PP	CXR, chest CT	HFNC, dexamethasone IV remdesivir IV, ceftriaxone IV, and IV azithromycin	Conservative	Transferred after 13 days
22	Suresh, et al. [26]	47	F	SLE, Sjogren’s syndrome, Hashimoto’s thyroiditis, and GERD	SOB, nausea, and vomiting	Yes, after diagnosis	PM and PP	CXR and chest CT	HFNC, remdesivir IV, dexamethasone IV, vancomycin, piperacillin-tazobactam, heparin IV	Chest tube	Died after 25 days
23	Suresh, et al. [26]	33	M	Asthma	Worsening SOB	Yes, after diagnosis	PM	CXR and chest CT	NIV, remdesivir, methylprednisolone, azithromycin, ceftriaxone, tocilizumab, dexamethasone, heparin infusion	Conservative	Transferred after 10 days
24	Machiraju, et al. [27]	51	M	HTN and DM	8 days of fever, myalgia, and dry cough	Yes, before diagnosis	PM, PP	CXR and chest CT	Meropenem, Targocid, and itraconazole.	Conservative	Died after 10 days
25	Machiraju, et al. [27]	77	M	HTN	7 days of SOB	Yes, before diagnosis	PM, PP	CXR and chest CT	High-flow oxygen, dexamethasone, LMWH, remdesivir, meropenem, Targocid	Conservative	Died after 17 days
26	Machiraju, et al. [27]	53	F	DM and dyslipidemia	5 days of fever and headache	No	PM	CXR and chest CT	Oxygen via nasal prongs, dexamethasone, remdesivir, LMWH	Conservative	Recovered after 15 days
27	Kalpaxi, et al. [28]	49	M	obesity	Fever, chest pain, and dry cough	No	PM	Chest CT	HFNC, corticosteroid, LMWH antibacterial, antiviral (not specified)	Conservative	Recovered after 22 days
28	Kalpaxi, et al. [28]	66	M	HTN, dyslipidemia, and obesity	3 days of fever, dyspnea, and dry cough	Yes, after diagnosis	PM	Chest CT	Oxygen via Venturi mask, corticosteroid, LMWH, antibacterial, and antiviral (not specified)	Conservative	Recovered after 30 days
29	Kalpaxi, et al. [28]	44	M	NKC	5 days of fever, dyspnea, and dry cough	No	PM and PP	Chest CT	HFNC, corticosteroid, LMWH antibacterial, antiviral (not specified)	Conservative	Recovered after 18 days
30	Chowdhary, et al. [29]	71	M	DM and HTN	8 days of fever, productive cough, and SOB	No	PM	CXR and chest CT	BiPAP, remdesivir, LMWH, clarithromycin, dexamethasone, ivermectin, zinc, pirfenidone acetylcysteine, piperacillin and tazobactam, methylprednisolone, nebulization of levosalbutamol, ipratropium, and budesonide	Conservative	Recovered after 6 days
31	Chowdhary, et al. [29]	61	M	DM and HTN	9 days of fever, non-productive cough, SOB, and weakness	Yes, after diagnosis	PM and PP	CXR and chest CT	NIV, remdesivir, meropenem, LMWH, doxycycline, dexamethasone, ivermectin, zinc, pirfenidone, nebulization of levosalbutamol, ipratropium, and budesonide	Conservative	Died after 3 days
32	Chowdhary, et al. [29]	30	M	NKC	5 days of fever, productive cough, and SOB	No	PM	Chest CT	NIV, remdesivir, meropenem, LMWH, doxycycline, dexamethasone, ivermectin, zinc, nebulization of levosalbutamol, ipratropium, and budesonide	Conservative	Recovered after 16 days
33	Protrka, et al. [30]	56	F	Chronic lymphocytic leukemia	7 days of fever, cough, SOB, headache, and nausea	No	PM	CXR and chest CT	Supplemental oxygen, azithromycin, corticosteroids, LMWH, IV paracetamol	Conservative	Recovered after 37 days
34	Protrka, et al. [30]	67	M	HTN	Fever, cough, and dyspnea	No	PM	CXR and chest CT	Oxygen mask, LMWH, corticosteroids, antibiotics (not specified)	Thoracic drainage catheter	Died after 14 days
35	Protrka, et al. [30]	74	M	HTN, prostate and bladder cancer	10 days of fever, SOB, and cough	No	PM	CXR and chest CT	HFNC, LMWH, corticosteroids, antibiotics (not specified)	Thoracic drainage catheter	Recovered after 30 days
36	Kooblall, et al. [31]	41	M	Asthma and obesity	Myalgia, pyrexia, dyspnea, and cough	No	PM	CXR and chest CT	CPAP, tocilizumab, steroids, and antibiotics (not specified)	Conservative	Recovered after 21 days
37	Kooblall, et al. [31]	52	M	Asthma	Dyspnea and chest pain	No	PM	CXR and chest CT	CPAP, tocilizumab, steroids, and antibiotics (not specified)	Conservative	Recovered after 2 days
38	Kong, et al. [32]	62	M	Bronchitis	14 days of fever, dry cough, SOB, wheezing, myalgia, nausea, and vomiting	No	PM	Chest CT	Non-rebreather mask, gamma globulin, dexamethasone, doxofylline, and nikethamide. Methylprednisolone, antibacterial, and antiviral (not specified)	Conservative	Recovered after 12 days
39	Urigo, et al. [33]	54	M	HTN and DM	10 days of cough and 4 days of SOB	No	PM	CXR and chest CT	Oxygen nasal cannula	NA	Died after 2 hours
40	Chaudhry, et al. [34]	52	M	Morbid obesity, allergies, and HTN	7 days of fever, malaise, cough, and dyspnea	No	PM	CXR and chest CT	HFNC, steroids, IL-6 inhibitors, plasma, remdesivir	Conservative	Recovered after 5 weeks
41	Yiğit, et al. [35]	23	M	Asthma	7 days of cough, SOB, and chest pain	No	PM	Chest CT	Low-flow nasal oxygen, favipiravir, LMWH, prednisolone, and levofloxacin	Conservative	Recovered
42	Hayrabedian, et al. [36]	59	M	NA	NA	Yes, before diagnosis	PM	Chest CT	Airway pressure release ventilation	Infraclavicular incisions	Died after 13 hours
43	Pooni, et al. [37]	56	M	DM, HTN, and seasonal asthma	14 days of a non-productive cough, dyspnea, and fever	No	PM	CXR and chest CT	CPAP, antibiotic (co-amoxiclav and clarithromycin) which was escalated to piperacillin, and tazobactam on the fifth day	Conservative	Recovered after 18 days
44	Malekpour, et al. [38]	54	M	HTN and previous right kidney transplant surgery	7 days of dry cough, SOB, nausea, vomiting, and decreased appetite	No	PM	Chest CT	Ceftriaxone, remdesivir, and methylprednisolone	Conservative	Recovered after 6 days
45	Malekpour, et al. [38]	71	M	Hypothyroidism	3 days of fever, dry cough, headache, and sore throat	No	PM	Chest CT	Ceftriaxone and levofloxacin	Conservative	Recovered after 5 days
46	Ramavath, et al. [39]	45	M	NKC	7 days of bilateral chest and neck and upper limb swelling	No	PM	CXR and chest CT	NA	Conservative	Recovered after 7 days
47	Ramezani, et al. [40]	43	M	NKC	4 days of cough, headaches, myalgia, fatigue, and SOB	No	PM and PP	Chest CT	Oxygen mask, imipenem, omeprazole, chloroquine phosphate, lopinavir, ribavirin, hydrocortisone, IV vitamin C	Conservative	Recovered after 14 days
48	Heijboer, et al. [41]	74	M	NKC	7 days of fatigue, loss of appetite, cough, and SOB	No	PM and PP	CXR and chest CT	HFNC, dexamethasone, dalteparin	Conservative	Recovered after 9 days

Table 2 summarizes the cases of pneumomediastinum and/or pneumopericardium found in large cohorts and case series studies. Different countries around the world have documented the occurrence of these complications in the literature. The number of cases reported in each county varied between 4 and 34 cases. Interestingly, India has the highest reporting rate among all countries.

**Table 2:** Prevalence of pneumomediastinum and pneumopericardium among COVID-19 patients in cohorts and case series studies.

Reference	Type of study	Prevalence, n (%)	Place
Juárez-Lloclla, et al. [42]	Case series study	12	Peru
Mart, et al. [43]	Cohort study	5 (5.4%)	Tennessee
Wali, et al. [44]	Case series study	5	United Kingdom
Cut, et al. [45]	Case series study	11	Romania
Haberal, et al. [46]	Cohort study	7 (0.02%)	Finland
Hamouri, et al. [47]	Cohort study	15 (0.81%)	Jordan
Kumar, et al. [48]	Cohort study	15 (7.56%)	India
Loffi, et al. [49]	Cohort study	6 (5.56%)	Italy
Kabi, et al. [50]	Case series study	4	India
Gorospe, et al. [51]	Case series study	4	Iran
Gandolfo, et al. [52]	Case series study	11	Italy
Adhikary, et al. [53]	Case series study	12	India
Kangas-Dick, et al. [54]	Cohort study	34 (10%)	United States
Miyakawa, et al. [55]	Cohort study	13	United States
Agrawal, et al. [56]	Case series study	4	India

Discussion

*Etiology* 

Many predisposing factors have been associated with SPM. They include cough, obesity, preexisting lung disease, and Valsalva maneuver. However, most are idiopathic [57-59]. The pathophysiology of SPM and PP is explained by the Macklin phenomena. It asserts that air following alveolar rupture dissects through the peri-bronchial vascular sheath and into the mediastinum and pericardium, leading to SPM and PP, respectively [60]. Alveolar rupture may result from a large pressure gradient between the marginal alveoli and the lung interstitium [54].

Currently, no obvious pathophysiological etiology of PM and PP in COVID-19 patients exists. Although PM and PP occurred in almost all age groups, most of the cases (73%) were above 50 years old (Table 1), suggesting that age may play a role in this condition. Furthermore, most of the reported cases were male patients (83.3%) (Table 3), raising the possibility that gender might be a predisposing factor. The number of reported cases is still small, and a comprehensive epidemiological study would be necessary to draw definitive conclusions. Hypertension, diabetes, asthma, and obesity were common comorbidities in many cases. Hypertension was the most common, indicating that it might be a predisposing factor. SPM may also occur following cytokine storm-induced diffuse alveolar damage that increases the probability of alveolar rupture [44]. The use of mechanical ventilation with high positive end-expiratory pressure (PEEP) was suggested to develop PM and PP in COVID-19 patients [17,47]. 

**Table 3:** Summary of case reports included in this review. CT, computed tomography; CXR, chest x-ray; PM, pneumomediastinum; PP, pneumopericardium.

Variable		Frequency	%
Gender	Male	40	83.33%
	Female	8	16.66%
Intubation requirement	Not required	27	56.25%
	Required before diagnosis	10	20.83%
	Required after diagnosis	11	22.92%
Diagnosis	PM	25	52.08%
	PP	2	4.16%
	PM and PP	21	43.75%
Radiological modality	Chest CT only	14	29.16%
	CXR only	3	6.25%
	Chest CT and CXR	31	64.58%
Outcome	Died	15	31.25%
	Recovered	31	64.58%
	Transferred to another care-center	2	4.16%

Imaging Modalities

In imaging, COVID-19 pneumonia appears as multifocal opacities with a ground-glass appearance (GGO) [61]. Most often, it appears bilaterally in the peripheral and lower area of the lung [5]. The chest x-ray (CXR) is the initial imaging study in patients with COVID-19 due to its availability and affordability. Furthermore, it is the only imaging method available for critically ill patients in the ICU [62]. Factors such as poor inspiration and positioning may lead to inaccurate interpretation [63]. In the event of unexplained clinical deterioration in COVID-19 patients, imaging is essential to diagnose possible complications such as PM and PP [37]. Chest computed tomography (CT) is a valuable tool in screening for complications and ruling out coexisting conditions [28]. The most common CT finding is peripheral, multifocal, bilateral, and GGO with or without consolidation. In addition, cavitation, nodule formation, interlobular septal thickening, and pleural effusion can be seen in rare cases [64]. Moreover, CT can detect the Macklin effect, which helps diagnose PM and PP [60].

Management and Treatment

The mainstay of managing hospitalized COVID-19 patients is monitoring the vital signs and providing symptomatic relief. The use of corticosteroids should be reserved for patients receiving supplemental oxygen or those on ventilator support. In addition, remdesivir is recommended for severe cases or for those who have a high risk of disease progression. Immunomodulatory drugs, such as baricitinib or tocilizumab, can be considered for patients with rapidly declining oxygen levels [65]. The decision to start empiric antibiotic therapy may be considered sometimes, as it is difficult to clinically distinguish between COVID-19 infection and community or hospital-acquired bacterial pneumonia. Further, if bacterial pneumonia co-infection is suspected, starting empirical antibiotic therapy is also recommended [66,67].

Most cases of PM and PP are benign and self-limiting [9]. Nonetheless, close monitoring is always recommended due to the possibility of serious circulatory and respiratory complications [20]. Behzadnia et al. [15] reported a case of concurrent PM and PP that required ICU admission. Despite the unfavorable progression, they successfully managed their patient with conservative measures only. To date, no clinical guidelines have been developed for the management of PM and PP in COVID-19 patients. For this reason, physicians must determine the appropriate management approach on a case-by-case basis.

The literature describes several approaches to managing PM in COVID-19 patients. In several cases, conservative management was sufficient in treating patients (Table 1). Surgical decompression was required for tension PM via thoracotomy [10]. Li et al. [50] reported two cases where they used a pigtail catheter to decompress the mediastinum. Moreover, Wali et al. [44] used subcutaneous and intrapleural chest drains. In the retrospective study by Kangas-Dick et al. [54], there was no significant difference in the mortality rates between patients who were managed conservatively or by surgical measures. For this reason, a conservative approach is recommended to be the mainstay of management in the absence of compressive symptoms. Patients with asymptomatic PP can be managed conservatively, while those with tension PP should be managed with a pericardial aspiration to avoid possible hemodynamic deterioration. A drainage tube placement into the pericardial sac may be necessary if the PP is persistent or recurring [19]. Ghods et al. reported a case of tension PP that required emergency echocardiography-guided catheter placement [11].

The use of mechanical ventilation with high PEEP was hypothesized to be one of the etiologies for developing PM and PP in COVID-19 patients [14]. A recent case series described five patients with COVID-19 pneumonia who developed PM shortly after being mechanically ventilated [44]. Singh et al. [9] avoided the use of mechanical ventilation to prevent further barotrauma. Interestingly, their patient achieved full recovery after six days with only supplemental oxygen. To reduce the risk of barotrauma in mechanically ventilated patients, physicians are challenged with the dilemma of achieving optimal oxygenation in the lowest ventilatory setting possible. Strategies to lower the required PEEP settings, such as treating reversible causes and applying for a proning position, should be considered [25]. In their case series, Suresh et al. [26] applied a protocol that involves prone positioning for 18 hours a day to enhance oxygenation in their patients. Interestingly, frequent proning in mechanically ventilated patients was suggested to cause barotrauma, leading to the development of PM [44]. These findings warrant further investigation to determine the safety of such intervention.

Prognosis

Out of 48 patients in our review, 40 (83.3%) were male. It is not clear if this disparity is because of the relatively few reported cases or if gender predisposes the development of such pathologies. Further, 72.9% (35 patients) had chronic illnesses that varied from diabetes mellitus, hypertension, heart failure, chronic kidney disease, obesity, asthma, and drug abuse. The incidence of PM and PP in COVID-19 patients is still unknown. Multiple studies with a relatively small-sized population reported widely variable results between 0.02% and 10% (Table 2). While PM is considered a benign condition, its occurrence in patients with SARS infection resulted in worse outcomes, with 38% requiring intubation and a 31% mortality rate [68]. In COVID-19 patients, developing PM is a bad prognostic indicator, indicative of extensive damage to the alveoli [49]. The total mortality rate in our review was 31.25% (15 patients), while 64.58% (31 patients) achieved full recovery, and 4.16% (2 patients) were transferred to another care facility for further management (Table 3).

Air leaks can occur spontaneously, as seen in COVID-19 patients, or secondary to barotrauma from mechanical ventilation [17]. In the present review, more than half of the cases occurred spontaneously, without mechanical ventilation. Barotrauma increases the risk of mortality and length of hospital stay [27]. Its prevalence in mechanically ventilated COVID-19 patients was 15%, compared to 0.5% in mechanically ventilated patients for other etiologies [69]. Thus, COVID-19 infection on its own can predispose individuals to develop barotrauma. Patients with COVID-19 pneumonia who are mechanically ventilated have a poor prognosis, with estimated mortality rates between 40% and 60.4% [70,71]. Mechanical ventilation can lead to severe complications, namely tension pneumomediastinum and tension pneumopericardium, which can result in a rapid deterioration of hemodynamic status [6].

## Conclusions

Patients with COVID-19 may experience unusual complications, such as PM and PP. Our literature review identified 48 cases. Various clinical presentations were associated with these complications; however, radiological findings were predominantly similar in all cases. PM and PP have been reported in nearly all age groups, but most patients were older than 50 years and mostly males. Clinical outcomes were influenced by comorbid conditions such as hypertension, diabetes, asthma, and obesity. Several surgical approaches have been described in the literature for managing PM and PP; however, most patients had a full recovery with only conservative measures.

Identifying and treating these complications promptly is essential to improve the survival rates. Small numbers of case reports and cohort studies have been published on this topic; more research is needed to determine the pathophysiology and risk factors and to establish evidence-based management guidelines.
